# miR-145 as a Potential Biomarker and Therapeutic Target in Patients with Non-Small Cell Lung Cancer

**DOI:** 10.3390/ijms241210022

**Published:** 2023-06-12

**Authors:** William C. Cho, Chi F. Wong, Kwan P. Li, Alvin H. Fong, King Y. Fung, Joseph S. Au

**Affiliations:** 1 Department of Clinical Oncology, Queen Elizabeth Hospital, Hong Kong SAR, China; 2 Oncology Center, Hong Kong Adventist Hospital, Hong Kong SAR, China

**Keywords:** biomarker, *GOLM1*, microRNA (miRNA), miR-145, non-small cell lung cancer (NSCLC), *RTKN*

## Abstract

Our previous study found that miR-145 was downregulated in non-small cell lung cancer (NSCLC) tissues and that it could inhibit the cell proliferation in transfected NSCLC cells. In this study, we found that miR-145 was downregulated in NSCLC plasma samples compared to healthy controls. A receiver operating characteristic curve analysis indicated that plasma miR-145 expression was correlated with NSCLC in patient samples. We further revealed that the transfection of miR-145 inhibited the proliferation, migration, and invasion of NSCLC cells. Most importantly, miR-145 significantly delayed the tumor growth in a mouse model of NSCLC. We further identified *GOLM1* and *RTKN* as the direct targets of miR-145. A cohort of paired tumors and adjacent non-malignant lung tissues from NSCLC patients was used to confirm the downregulated expression and diagnostic value of miR-145. The results were highly consistent between our plasma and tissue cohorts, confirming the clinical value of miR-145 in different sample groups. In addition, we also validated the expressions of miR-145, *GOLM1*, and *RTKN* using the TCGA database. Our findings suggested that miR-145 is a regulator of NSCLC and it plays an important role in NSCLC progression. This microRNA and its gene targets may serve as potential biomarkers and novel molecular therapeutic targets in NSCLC patients.

## 1. Introduction

According to GLOBOCAN 2020 estimates, lung cancer is the second most common cancer (11.4%) and the leading cause of cancer death (18%) worldwide [1]. About 80% to 85% of lung cancers are non-small cell lung cancers (NSCLC). The major subtypes of NSCLC are adenocarcinoma, squamous cell carcinoma, and large cell carcinoma. During the progression of lung cancer, tumor cells can migrate and invade in various ways. The different mechanisms of cancer development and metastasis are complex and many players are known to be involved in tumorigenesis, including microRNA (miRNA) [2].

miRNAs are evolutionarily conserved, endogenous, small non-coding RNA molecules, approximately 22 nucleotides in length, which function as post-transcriptional gene regulators. Many miRNAs have been identified as oncogenes, tumor suppressors, and even regulators of cancer stemness and metastasis. These miRNAs are emerging as important regulators in cellular pathways and they appear to play key roles in tumorigenesis [3]. Accumulating evidence indicates that miRNAs are aberrantly expressed in lung cancer and that miRNA dysfunction plays a crucial role in tumor developmental strategies. Therefore, further understanding the role of miRNAs in the progression of lung cancer and its related molecular mechanisms is of great value for the development of new diagnostic and therapeutic strategies.

Our previous study showed that miR-145 functions as a tumor suppressor in NSCLC tissues and the restoration of miR-145 suppresses cancer cell growth in *EGFR*-mutated NSCLC patients [4]. miR-145 is located on chromosome 5 (5q32-33) and was first reported in mouse myocardium and later found in humans [5,6,7]. It is prominently expressed in germline and mesoderm-derived tissues, including the heart, ovaries, prostate, spleen, testes, and uterus [8]. miR-145 plays a profound role in the initiation and progression of various tumors. This miRNA not only regulates tumor growth, invasion, and metastasis, but is also important for tumor angiogenesis and the proliferation of cancer stem cells. It is a tumor suppressor that targets various tumor-specific genes and proteins, thereby affecting their related signaling pathways [9]. It has been reported that miR-145 is downregulated in several cancers, including lung cancer [10]. We further demonstrated that miR-145 inhibits cell proliferation in NSCLC by targeting *EGFR* and *NUDT1* [11]. Our findings identified the tumor-suppressive role of miR-145 in NSCLC and revealed potential therapeutic strategies for NSCLC treatment. However, the role of miR-145 in NSCLC progression and its related molecular mechanisms remains largely unknown. This study aims to investigate how miR-145 functions as a tumor suppressor in NSCLC and whether there is any direct gene target of miR-145 in NSCLC.

## 2. Results

### 2.1. miR-145 Expression Is Downregulated in the Human Plasma Samples of NSCLC Patients

**Figure 1:**
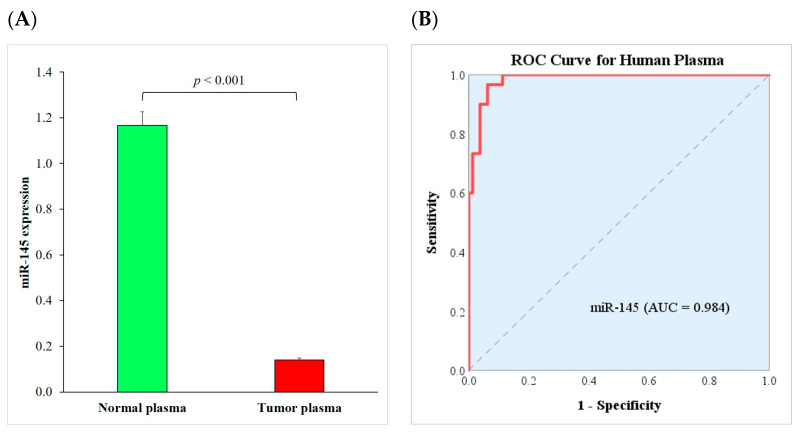
Downregulation of miR-145 in NSCLC patient plasma. (**A**) Expression levels of miR-145 were significantly downregulated in NSCLC plasma (*N* = 80) compared to normal plasma (*N* = 30) measured using RT-qPCR. Each experiment was performed in triplicate and data are shown as mean ± standard error of the mean. (**B**) ROC curve analysis shows that miR-145 can distinguish NSCLC patients from healthy controls in plasma samples. AUC, area under the ROC curve; NSCLC, non-small cell lung cancer; RT-qPCR. reverse transcription quantitative real-time polymerase chain reaction; and ROC, receiver operating characteristic.

To investigate whether miR-145 plays a role in NSCLC, we first analyzed the expression of miR-145 in the plasma of 80 NSCLC patients and 30 healthy donors. We found that the expression of miR-145 was significantly downregulated in the NSCLC plasma (0.141 ± 0.020) compared to the normal plasma (1.167 ± 0.137) (*p* < 0.001) (Figure 1A). We then evaluated the correlation between miR-145 expression and the clinical characteristics of NSCLC patients. As shown in Table 1, the expression of miR-145 was significantly different between the early-stage (I and II) and advanced-stage (III and IV) patients (*p* = 0.013). However, the expression of miR-145 was not significantly different among patients with different ages, sexes, smoking histories, and overall survival. To elucidate the diagnostic value of circulating miR-145 in NSCLC patients, a receiver operating characteristic (ROC) curve analysis was performed. We found that miR-145 could distinguish NSCLC patients from healthy controls with an area under the ROC curve (AUC) of 0.984 (Figure 1B). This high AUC value suggested that the downregulation of plasma miR-145 levels might help to identify NSCLC patients from healthy subjects with a high sensitivity and specificity. These results suggest that plasma miR-145 levels are correlated with NSCLC.

### 2.2. Upregulated miR-145 Expression Inhibits Proliferation, Migration, and Invasion of NSCLC Cells

**Figure 2:**
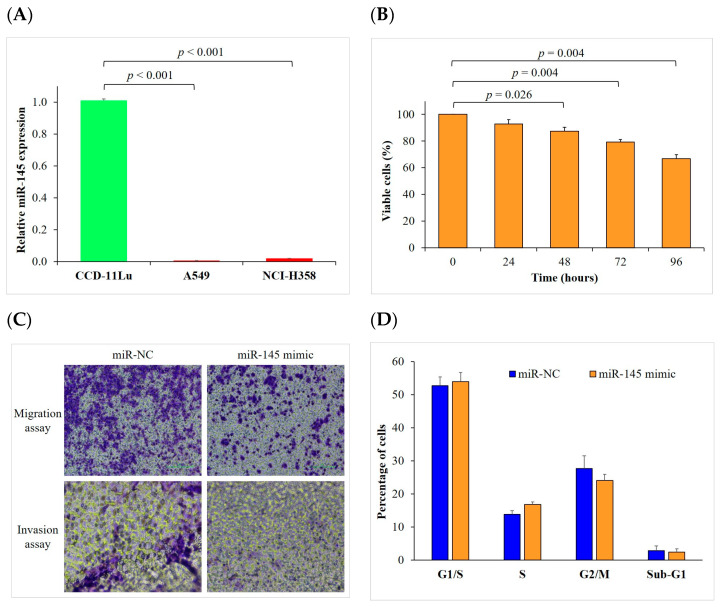
Upregulated miR-145 inhibits cell proliferation, migration, and invasion. (**A**) The relative expression of miR-145 was significantly lower in NSCLC cell lines (A549 and NCI-H358) when compared to a normal human lung fibroblast cell line (CCD-11Lu) measured using RT-qPCR. (**B**) miR-145 mimic transfection significantly inhibited cell proliferation in NCI-H358 cells at the last three time points (*p* = 0.026, 0.004, and 0.004 vs. pre-transfection, respectively). (**C**) miR-145 inhibits NSCLC migration in A549 cells 24 h after transfection with miR-145 mimic. miR-145 also inhibits NSCLC invasion in A549 cells 48 h after transfection with miR-145 mimic. (**D**) Flow cytometry analysis was used to examine the relative cell cycle composition of NCI-H358 cells transfected with miR-145 mimic or miR-NC, our results showed no significant difference between miR-145 and controls. Each experiment was performed in triplicate and data are shown as mean ± standard error of the mean. NC, negative control; NSCLC, non-small cell lung cancer; and RT-qPCR, reverse transcription quantitative real-time polymerase chain reaction.

To reveal the expression levels of miR-145 in NSCLC, we measured the expressions of miR-145 in two human NSCLC cell lines (A549 and NCI-H358) and a normal human lung fibroblast cell line (CCD-11Lu) using reverse transcription quantitative real-time polymerase chain reactions (RT-qPCR). The relative expression of miR-145 was significantly lower in the two NSCLC cell lines when compared to the CCD-11Lu cells (*p* < 0.001) (Figure 2A). We then tested the effect of miR-145 on the tumor activity. To assess whether the upregulation of miR-145 is involved in NSCLC progression in vitro, we first investigated whether miR-145 expression affected the proliferation of lung cancer cells. Compared to the pre-transfected cells, we found that an overexpression of miR-145 significantly (*p* = 0.026, 0.004, and 0.004, respectively) inhibited the proliferation of the NCI-H358 cells (87% ± 3%, 79% ± 2%, and 67% ± 3%, respectively) at the last three time points (48, 72, and 96 h). The viability of the cells was reduced in a time-dependent manner via transfection with an miR-145 mimic (Figure 2B). We then examined whether miR-145 expression altered tumor cell migration and invasion, which are critical for tumor metastasis. Using a transwell migration assay, we transfected the A549 cells with an miR-145 mimic or its negative control (miR-NC) and found that cell migration was significantly inhibited by the miR-145 mimic transfection. We also showed that the transfection of an miR-145 mimic into the A549 cells significantly inhibited cell invasion using a transwell invasion assay (Figure 2C). These results suggest that miR-145 plays an inhibitory role in cell proliferation, migration, and invasion. To determine whether the cell cycle is associated with miR-145, we assessed the effect of an miR-145 mimic transfection on the cell cycle of the NCI-H358 cells using flow cytometry. Our results indicated that the upregulated expression of miR-145 did not show significant effects on the cell cycle (Figure 2D).

### 2.3. miR-145 Overexpression Delays Tumor Growth in a Nude Mouse Model

**Figure 3:**
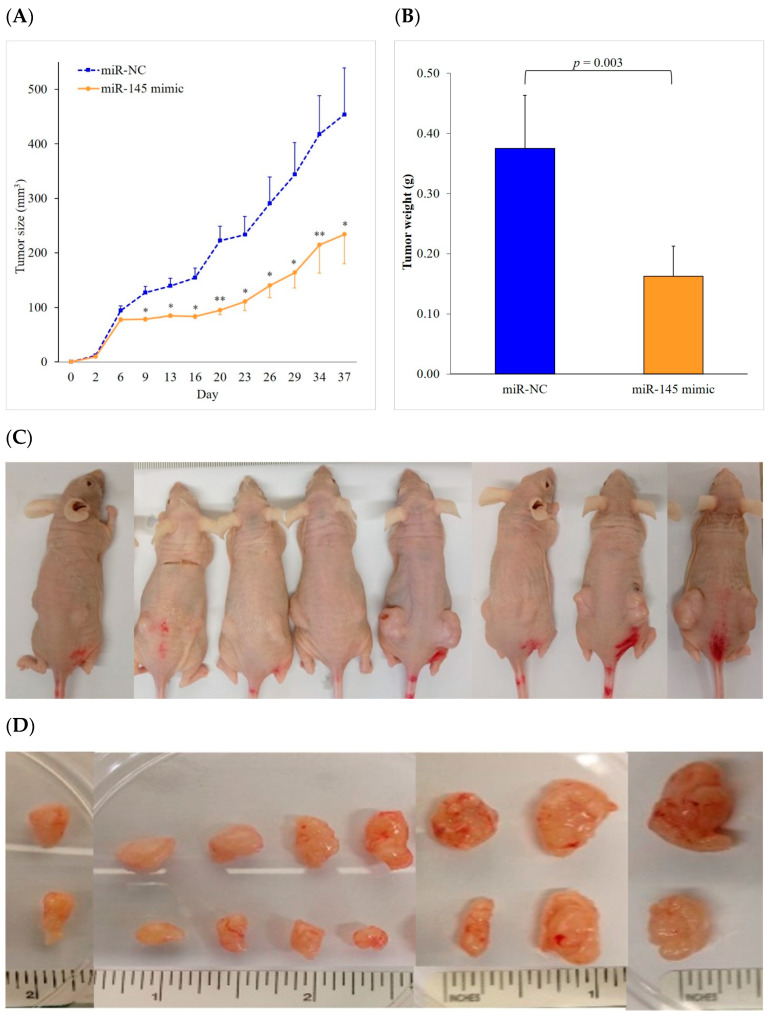
miR-145 transfection delays tumor growth in a mouse model of NSCLC. (**A**,**B**) NCI-H358 human NSCLC cells transfected with miR-145 mimic or miR-NC were injected subcutaneously into the left or right flank of eight BALB/c nude mice, respectively. After 37 days, animals were sacrificed and solid tumors were dissected. Transfection of miR-145 mimic showed reduced tumor size (48% smaller) and tumor weight (57% lighter) than miR-NC. * *p* < 0.005, ** *p* < 0.0005. (**C**) Visible tumors at the injection site: tumor transfected with miR-145 on the left flank and tumor transfected with miR-NC on the right flank. (**D**) The lower row is the tumor transfected with the miR-145 mimic, while the upper row is the tumor transfected with miR-NC. NC, negative control; and NSCLC, non-small cell lung cancer.

To examine the effect of miR-145 on tumor growth in vivo, H358-miR-145 mimic and H358-miR-NC cells were injected into the left and right flank subcutaneous tissues of eight BALB/c nude mice, respectively. After 37 days, the tumors transfected with the miR-145 mimic had grown significantly slower than those transfected with the miR-NC (*p* < 0.005). The tumor growth was delayed by 48% after the transfection with the miR-145 mimic (234 ± 54) compared to that with the miR-NC (454 ± 86) (Figure 3A). The tumors transfected with the miR-145 mimic (0.16 ± 0.05) were also lighter (*p* = 0.003) than those transfected with the miR-NC (0.38 ± 0.09). The transfection with the miR-145 mimic resulted in a 57% lighter tumor weight than that with the miR-NC (Figure 3B). Figure 3C,D show the appearance of the tumors in the nude mice, where the tumors transfected with the miR-145 mimic were smaller. Our results suggest that miR-145 delays tumor growth in vivo, which is consistent with our in vitro cell proliferation results.

### 2.4. miR-145 Downregulates the Enzyme Activities of Golgi Phosphoprotein 73 (GOLM1) and Rhotekin (RTKN) through Their 3′-UTRs

**Figure 4:**
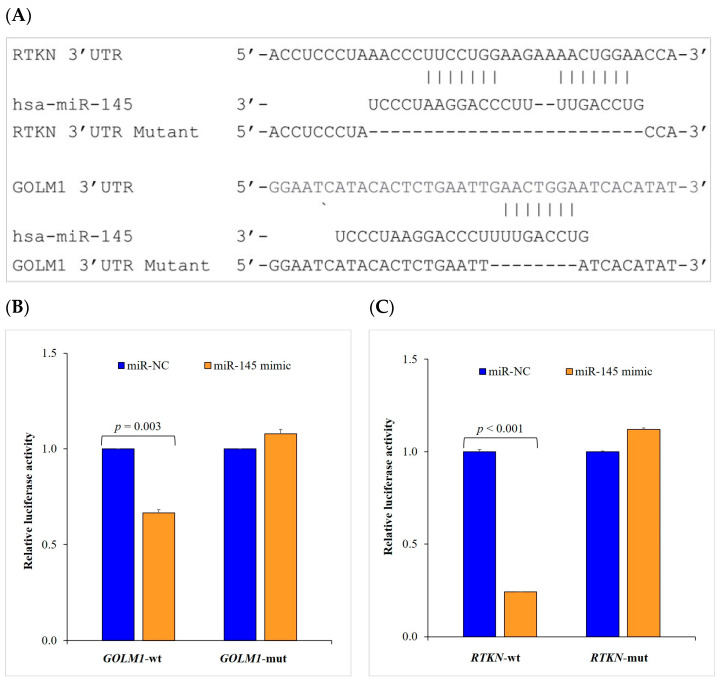
miR-145 targets *GOLM1* and *RTKN* in NSCLC cells. (**A**) In silico analysis of miR-145 binding sites in *GOLM1* and *RTKN* 3′-UTR. (**B**,**C**) *GOLM1* and *RTKN* 3′-UTR luciferase reporter plasmids (*GOLM1*-wt and *RTKN*-wt) or their mutant plasmids (*GOLM1*-mut and *RTKN*-mut) in the presence of miR-145 mimic or miR-NC were transfected into A549 human NSCLC cells. Each experiment was performed in triplicate and relative luciferase expressions are shown as mean ± standard error of the mean. 3′-UTR, 3′-untranslated region; mut, mutant; NC, negative control; NSCLC, non-small cell lung cancer; and wt, wild-type.

To further investigate the tumor-suppressive role of miR-145, we sought to identify the relevant endogenous targets of miR-145. We used two target prediction programs (miRSearch 3.0 and miRTarBase) to screen for miR-145 targets, in which two oncogenes (*GOLM1* and *RTKN*) were identified as the putative targets of miR-145 (Figure 4A). To assess whether *GOLM1* and *RTKN* are regulated by miR-145, we generated wild-type (wt) *GOLM1* and *RTKN* 3′-UTR luciferase reporter plasmids (*GOLM1*-wt and *RTKN*-wt) and their mutant plasmids (*GOLM1*-mut and *RTKN*-mut). We then analyzed the effect of miR-145 on the reporter gene activity in the A549 cells. Our data showed that miR-145 significantly reduced the luciferase activity of *GOLM1*-wt (1.000 ± 0.000 vs. 0.665 ± 0.018, *p* = 0.003) and *RTKN*-wt (1.000 ± 0.011 vs. 0.242 ± 0.001, *p* < 0.001), but not *GOLM1*-mut and *RTKN*-mut (Figure 4B,C). These data suggest that *GOLM1* and *RTKN* are direct targets of miR-145.

### 2.5. The Expression and Clinical Value of miR-145 and Its Gene Targets in Paired Lung Tissues of NSCLC Patients

**Figure 5:**
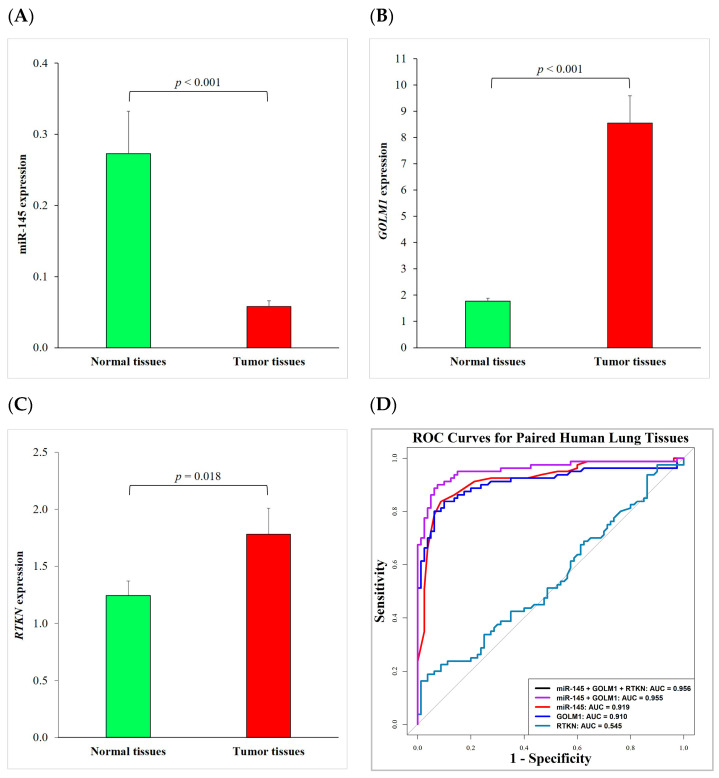
The expression and clinical performance of miR-145 and its gene targets in paired lung tissues of NSCLC patients. (**A**–**C**) The expression levels of miR-145, *GOLM1*, and *RTKN* were measured using RT-qPCR in paired tumors and their adjacent non-malignant lung tissues. (**D**) ROC curve analysis was used to measure the diagnostic performance of miR-145, *GOLM1*, and *RTKN* in paired lung tissues. ROC curves of the two-marker panel (miR-145 + *GOLM1*) and three-marker panel (miR-145 + *GOLM1* + *RTKN*) were almost overlapped. Each experiment was performed in triplicate and data are shown as mean ± standard error of the mean. AUC, area under the ROC curve; NSCLC, non-small cell lung cancer; RT-qPCR, reverse transcription quantitative real-time polymerase chain reaction; and ROC, receiver operating characteristic.

To confirm the interesting findings above, the expression levels of miR-145, *GOLM1*, and *RTKN* were measured using RT-qPCRs in a paired cohort of tumors and their adjacent non-malignant lung tissues from 80 NSCLC patients. We found that the expression of miR-145 in the tumor tissues (0.058 ± 0.008) was significantly lower than that in the adjacent normal tissues (0.273 ± 0.060) (*p* < 0.001) (Figure 5A). In contrast, the expressions of *GOLM1* and *RTKN* in the tumor tissues (8.549 ± 1.034 and 1.782 ± 0.228, respectively) were significantly higher than those in the adjacent normal tissues (1.766 ± 0.113 and 1.247 ± 0.128, respectively) (*p* < 0.001 and *p* = 0.018) (Figure 5B,C). ROC curves were used to evaluate the diagnostic performances of miR-145, *GOLM1*, and *RTKN*. The AUCs were 0.919, 0.910, and 0.545, respectively (Figure 5D). These findings suggest that the expressions of miR-145 and its gene target *GOLM1* may serve as potential biomarkers for NSCLC, but indicate that *RTKN* has no diagnostic value in NSCLC tissues. These results indicate the diagnostic value of miR-145 and *GOLM1* in NSCLC tissues. We also conducted ROC curve analyses of a two-marker panel (miR-145 + *GOLM1*) and a three-marker panel (miR-145 + *GOLM1* + *RTKN*). Our results showed that the combined marker panels increased the AUCs to 0.955 and 0.956, respectively, suggesting a better performance of the combined marker panels at distinguishing between the tumor and adjacent normal tissues (Figure 5D).

### 2.6. External Validation in TCGA Data

**Figure 6:**
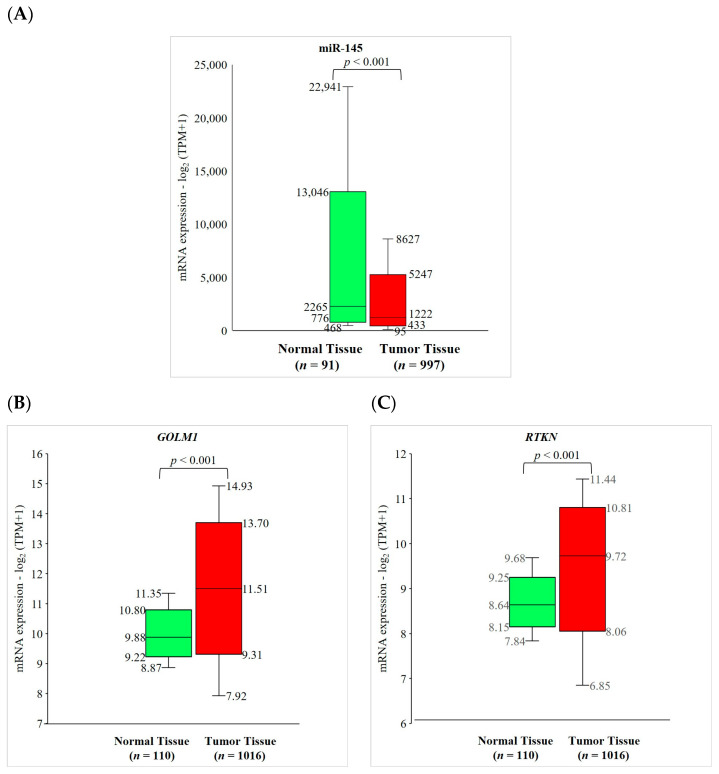
Validation of miR-145, *GOLM1*, and *RTKN* expressions using The Cancer Genome Atlas (TCGA) database. The expression of (**A**) miR-145 was significantly lower, while the expressions of (**B**) *GOLM1* and (**C**) *RTKN* were significantly higher in non-small cell lung cancer primary tumor tissue compared to solid normal tissue. mRNA, messenger RNA; and TPM, transcripts per million.

miR-145 and its two gene targets (*GOLM1* and *RTKN*) were then validated using the external TCGA database to confirm the validity of our findings. We found that the expression level of miR-145 was significantly lower (*p* < 0.001), while the expression levels of its two gene targets were significantly higher (*p* < 0.001) in NSCLC primary tumor tissues compared to solid normal tissues (Figure 6). These results confirm the cohort independence of our findings regarding the low expression of miR-145 and high expressions of *GOLM1* and *RTKN* in NSCLC patients.

## 3. Discussion

There are knowledge gaps in identifying and developing effective biomarkers that can detect lung cancer. To date, no single perfect biomarker has been found to meet the clinical needs for the optimal care of NSCLC patients. The identification of blood-based biomarkers may provide the non-invasive early detection and better stratification of high-risk patients in the future. A previous study has shown that specific miRNAs are good biomarker candidates for NSCLC [12]. In fact, miRNAs are very stable in liquid biopsies, making them ideal non-invasive biomarkers for cancer detection.

In the current study, we found that miR-145 expression is significantly downregulated in the plasma of NSCLC patients. In vitro studies showed that increasing miR-145 could inhibit the proliferation, migration, and invasion of NSCLC cells. We also showed that an miR-145 mimic can significantly delay tumor growth in vivo. Our findings in paired human tissues were consistent with those of the plasma results. Taken together, these experimental results reveal a negative regulatory relationship between miR-145 upregulation and NSCLC progression, highlighting the potential of miRNA-based NSCLC diagnosis and therapy.

miR-145 is a potent tumor suppressor downregulated in multiple cancers, including lung cancer [11], bladder cancer [13], breast cancer [14], hepatocellular carcinoma (HCC) [15], gastric cancer [16], and prostate cancer [17]. Several studies have shown that miR-145 can serve as a candidate for potentially repairing damaged DNA [18], inhibiting the proliferation of tumor cells [9], suppressing therapeutic resistance [19], and metastasis [20] by targeting various oncogenes. In recent years, the oncogenic roles of multiple signaling pathways in promoting carcinogenesis and tumor growth have received increasing attention. miR-145 has been reported to act as an important regulator of several well-documented pathways, such as the ERK/MAPK, mTOR/p70S6K1, and TGF-β pathways, which are frequently disrupted in cancer [21]. Zhou et al. [22] also showed that miR-145 inhibits chemoresistance by downregulating *AKT3* expression and further inhibiting the PI3K/AKT pathway.

Apart from lung cancer, accumulating evidence indicates that miR-145 is also involved in the pathogenesis of chronic obstructive pulmonary disease (COPD). Dang et al. [23] showed that miR-145-5p prevents cigarette smoke (CS) extract-induced apoptosis and inflammation in airway epithelial cells, in part by targeting *KLF5* in CS-mediated COPD. Gu et al. [24] found that the long non-coding RNA TUG1 promotes airway remodeling by inhibiting the miR-145-5p/DUSP6 axis in CS-induced COPD. Tiwari et al. [25] showed that miR-145-5p is associated with an early decline pattern in lung function growth, leading to COPD in children with asthma, and that it also increases the proliferation of airway smooth muscle cells. Furthermore, miR-145 has been shown to play a role in other lung diseases. McLendon et al. [26] demonstrated that antimiR-145 could reduce the extent of pulmonary arteriopathy and the severity of pulmonary hypertension. Cao et al. [27] showed that miR-145 negatively regulates the TGFBR2 signaling that contributes to sepsis-induced acute lung injury. On the other hand, an in vivo study of a lung ischemia/reperfusion injury mouse model revealed that miR-145 promotes NF-κB transcriptional activity through SIRT1 expression, thereby enhancing autophagy and aggravating lung ischemia/reperfusion injury [28].

Consistent with previous reports, our study confirmed that an overexpression of miR-145 inhibited the growth and metastasis of NSCLC cells. These pieces of evidence suggest that miR-145 has a correlation with NSCLC. Using a dual-luciferase assay, we demonstrated that *GOLM1* and *RTKN* are the direct targets of miR-145. We found that *GOLM1* and *RTKN* mRNAs were significantly downregulated by miR-145. The expression levels of miR-145 and its gene targets were validated using gene expression data from independent external NSCLC cohorts in the TCGA database.

GOLM1 is a glycosylated protein that is present on the cis-Golgi cisternae [29] and highly expressed in various cancers, including NSCLC [30]. It is one of the most promising markers for the early diagnosis and prognosis of HCC [31]. In recent years, GOLM1 has been identified as a multifunctional protein that promotes cancer progression and epithelial–mesenchymal transition in cancer cells [32]. In this study, we demonstrated that the upregulation of miR-145 suppresses the *GOLM1* expression in NSCLC cells. Their expressions show a strong negative correlation. A study found that the interaction of GOLM1 with the membrane protein EGFR played an important role in EGFR/RTK repurposing/stimulation and downstream MMP-9 expression by promoting HCC growth and metastasis [33]. Another study reported that the restoration of miR-145 in prostate cancer cells significantly inhibited cancer cell migration and invasion by directly targeting *GOLM1* [34]. Exploring the interaction between miR-145 and *GOLM1* in NSCLC samples may provide new insights into NSCLC tumorigenesis and improve therapeutic strategies for NSCLC patients.

The Rho family of GTPases is a subfamily of the Ras superfamily. Rho GTPases are key regulators of the cytoskeletal dynamics that affect many cellular processes, including cell polarity, migration, vesicle trafficking, and cytokinesis [35]. Rhotekin is an effector of Rho GTPase, a protein encoded by the *RTKN* gene [36]. An increasing number of studies have found that *RTKN* plays an important role in the development of many human cancers, including breast, colon, gastric, and lung cancers. miR-145 inhibits breast cancer cell growth by targeting *RTKN* [37]. A colon cancer cell analysis revealed that the long non-coding RNA HULC interacts with miR-613 to regulate colon cancer growth and metastasis by targeting *RTKN* [38]. *RTKN* can also act as an oncogene of gastric cancer by regulating the HDAC1/p53 pathway [39]. Studies on lung cancer cells have shown that *RTKN* inhibition exerts antitumor effects and that *RTKN* is associated with a sensitivity to HSP90 inhibitors [40,41]. In the current study, we found that an overexpression of miR-145 suppressed the expression of *RTKN*. This is the first time that *RTKN* has been identified as a direct target of miR-145 in NSCLC. Further research on this potential therapeutic target may help to develop treatment strategies for NSCLC patients.

Numerous reports have shown that the differential expression of miRNAs in tumor tissues, in comparison to non-malignant controls, may have diagnostic value for NSCLC [12]. Likewise, the circulating miRNAs in plasma are emerging as non-invasive biomarkers for NSCLC diagnoses [42]. Our ultimate goal is to develop an effective, non-invasive biomarker for detecting NSCLC. This study has potential implications for the identification of circulating miRNAs and their novel targets, which may be translated into future molecular markers and even therapeutic targets in NSCLC.

## 4. Materials and Methods

### 4.1. Patients’ Specimens

Plasma samples from 80 NSCLC patients (the median age was 64 years, ranging from 31 to 87 years, with 41 males and 39 females; 19 stage I, 19 stage II, 22 stage III, and 20 stage IV patients; 46 non-smokers and 34 smokers; the median overall survival was 42 months, ranging from 2 to 192 months), as well as paired tumors and their adjacent non-malignant lung tissues from 80 NSCLC patients, were retrieved from the archives of the Department of Clinical Oncology, Queen Elizabeth Hospital (Hong Kong). All the samples were collected from pretreatment patients that had been diagnosed with NSCLC. The plasma samples of normal controls were collected from 30 healthy donors with no evidence of lung cancer. The use of these samples was approved by the Cluster Research Ethics Committee of the Hospital Authority (Hong Kong SAR, China).

### 4.2. RNA Extraction and Reverse Transcription

Total RNA was extracted with TRIzol (Invitrogen, Carlsbad, CA, USA,) following the manufacturer’s instructions. For the gene expression analysis, cDNA was synthesized using SuperScript III reverse transcriptase (Invitrogen). For the miRNA expression analysis, cDNA was synthesized using the TaqMan MicroRNA Reverse Transcription Kit (Applied Biosystems, Foster City, CA, USA).

### 4.3. Quantitative Real-Time Polymerase Chain Reaction (qPCR)

TaqMan miRNA assays for miR-145 and normalized target RNU48 were purchased from Applied Biosystems. A QuantiNova Probe PCR Kit (Qiagen, Hilden, Germany) was used for the miRNA expression analysis. For the miR-145 expression, *RNU6* and miR-16 were used as endogenous reference genes in tissue samples and plasma samples, respectively. For the gene expression, the data were normalized to *HPRT1*. The primer sequences are listed in Table 2. A QuantiNova SYBR Green Kit (Qiagen) was used for the expression analysis. A qPCR was performed using a LightCycler 480 system (Roche, Switzerland). Each experiment was performed in triplicate. Relative quantification was performed using the ΔΔCt method.

### 4.4. Cell Culture

The human NSCLC cell lines (A549 and NCI-H358) and normal human lung fibroblast cell line (CCD-11Lu) were purchased from American Type Culture Collection (Manassas, MA, USA). A549 and CCD-11Lu were cultured in DMEM medium, while NCI-H358 was cultured in RPMI 1640 medium (Thermo Fisher Scientific Company, Branchburg, NJ, USA). Both mediums were supplemented with 10% fetal bovine serum (FBS) (Thermo Fisher Scientific Company) and 1% penicillin-streptomycin (Thermo Fisher Scientific Company). All the cell lines were cultured at 37 °C in a humidified incubator with 5% CO_2_. The cells were passaged using 0.25% trypsinization. The cells showing the fastest growth in the logarithmic phase of growth were selected for the experiments.

### 4.5. Cell Transfection

The miR-145 mimic and miR-NC were purchased from Invitrogen. The cells were transfected with miR-145 mimic or miR-NC using Lipofectamine RNAiMAX (Invitrogen), according to the manufacturer’s instructions. In brief, mirVana miRNA mimic hsa-miR-145-5p (Thermo Fisher Scientific Company) or mirVana miRNA mimic NC #1 (Thermo Fisher Scientific Company) was diluted to 30 μM and mixed with diluted Lipofectamine RNAiMAX reagent. After being incubated at room temperature for 5 min, the mixture was then added to the cells accordingly, and the plates were incubated at 37 °C for one day.

### 4.6. Cell Proliferation Assay

The transfected cells were seeded in 96-well plates at 3 × 10^3^ cells per well. The cell proliferation was measured with the cell proliferation reagent water-soluble tetrazolium salt (WST-1) (Roche, Germany) for 96 h. In each well of a 96-well tissue culture plate, approximately 4000 transfected cells were seeded and incubated at 37 °C for 24 h. Then, 100 µL of RPMI 1640 medium (Invitrogen) with 10% FBS and 10 µL of WST-1 reagent were added to each well. After being incubated at 37 °C for 2 h, the absorbance of the samples was measured using a spectrophotometer and monitored at intervals of 24 h, 48 h, 72 h, and 96 h. The results were then normalized with NC mimic. All the experiments were performed in triplicate.

### 4.7. Transwell Migration and Invasion Assays

The cell migration was assessed with a transwell migration assay. 1 × 10^5^ transfected cells were added to the upper chamber of the insert and allowed to migrate for 24 h. The cell invasion assays were performed with coating inserts with Matrigel. 1 × 10^5^ cells per well were allowed to infiltrate for 48 h. A 6.5 mm transwell (Corning, Steuben County, NY, USA) with 8.0 µm pore polycarbonate membrane inserts was used to help monitor the cell migration of the transfected cells compared to that of the controls. A medium containing 10% FBS was added to the wells of the plate, followed by the transwell inserts. Afterward, serum-free medium and the associated transfected cells were added to the inner compartment. The cells in the lower chamber were fixed and stained with crystal violet. All the experiments were performed in triplicate.

### 4.8. Flow Cytometry Analysis

The NCI-H358 human NSCLC cells were transfected with miR-145 mimic or miR-NC. After 48 h, the cells were harvested, washed with phosphate-buffered saline (PBS), and fixed overnight with 70% ice-cold ethanol. The fixed cells were washed with PBS and stained with 50 μg/mL of propidium iodide solution (Merck & Co, Rahway, NJ, USA) and 100 μg/mL of RNase A (Thermo Fisher Scientific Company). The stained cells were filtered through a 40 μm cell strainer (BD Biosciences, Franklin Lakes, NJ, USA) and analyzed using flow cytometry. The data were analyzed using BD FACSDiva v8.0.

### 4.9. Subcutaneous Tumor Xenograft Mouse Model

Eight 6- to 8-week-old BALB/c nude mice (20–25 g body weight) and NCI-H358 human NSCLC cells were used for the in vivo experiments. One million miR-145-mimic- or miR-NC-transfected NCI-H358 cells were injected subcutaneously into the left flank or right flank of these nude mice, respectively. The tumor growth was monitored twice weekly for one month. The maximum tumor diameters (x) and transverse diameters (y) of the tumors were measured. The tumor volume was calculated as follows: tumor volume (mm^3^) = (xy^2^)/2. After 37 days, the mice were sacrificed by isoflurane inhalation and the solid tumors were dissected and weighed.

### 4.10. Dual-Luciferase Reporter Gene Assay

The sequences containing the putative binding site for miR-145 in the 3′-untranslated region (UTR) of the wild-type (wt) *GOLM1* and *RTKN* and a mutant lacking the miR-145 binding site were inserted into the psiCHECK-2 vector (Promega, Fitchburg, WI, USA). A 100 ng wt vector and mutant vector, as well as 10 nM of miR-145 mimic and miR-NC, were co-transfected into the NSCLC cells using Lipofectamine 2000 (Invitrogen). After being transfected for 48 h, a luciferase assay was performed using the Dual-Luciferase Reporter Assay System (Promega), according to the manufacturer’s instructions. All the experiments were performed in triplicate.

### 4.11. The Cancer Genome Atlas (TCGA) Data Validation

The gene expression level from the RNA-seq data was downloaded from the TCGA ftp server. The normalized gene-level expression estimates were processed by adding a constant of 1 to all the expression estimates, followed by log_2_ transformation and median centering.

### 4.12. Statistical Analysis

The differences between the two independent groups were compared using the Mann–Whitney *U* test. The data from paired tissue samples were compared using a paired-sample *t*-test. All the results are expressed as means ± standard errors of the mean. The differential expression analysis of the TCGA data was performed using the Welch’s *t*-test using R (v4.2.0). Differences with a *p* value of less than 0.05 were considered to be statistically significant. An ROC analysis was used to evaluate the diagnostic value of miR-145 and its gene targets. All the analyses were performed using the SPSS software version 26 (SPSS Inc., Chicago, IL, USA). In addition, two target prediction programs (miRSearch 3.0 and miRTarBase) were used to screen for the miR-145 gene targets.

## 5. Conclusions

Our study confirmed that miR-145 expression is correlated with NSCLC in both plasma and tissue samples from NSCLC patients. miR-145 inhibits the proliferation, migration, and invasion of NSCLC cells, but does not affect the cell cycle regulation. It also leads to reduced tumor growth in mouse models. Most importantly, we identified two direct targets of miR-145 (*GOLM1* and *RTKN*), with *RTKN* being identified for the first time as an miR-145 target in NSCLC. In addition, we also validated the expressions of miR-145, *GOLM1*, and *RTKN* using the external TCGA database. Our findings suggest that miR-145 and its gene targets may serve as potential biomarkers and molecular therapeutic targets in NSCLC. As miR-145 has also been connected with COPD and other lung diseases, its potential as a diagnostic tool for lung cancer is unclear. Nevertheless, this study may improve our understanding of miR-145 as a tumor suppressor for NSCLC management. We also revealed some potential therapeutic targets for NSCLC treatment, but their clinical application warrants further investigation.

## Figures and Tables

**Table 1:** Correlation between plasma miR-145 expression and clinical characteristics of study subjects.

Characteristic	Case	Plasma miR-145 *	*p* Value
Age (years)			0.779
≤60	31	0.134 ± 0.031	
>60	49	0.145 ± 0.026	
Sex			0.906
Male	41	0.139 ± 0.030	
Female	39	0.143 ± 0.026	
Stage			0.013
Early (I and II)	38	0.093 ± 0.015	
Advanced (III and IV)	42	0.184 ± 0.034	
Smoking history			0.293
Non-smoker	46	0.159 ± 0.027	
Smoker	34	0.117 ± 0.029	
Overall survival			0.365
≥5 years	32	0.119 ± 0.027	
<5 years	48	0.155 ± 0.027	

* Data are shown as mean ± standard error of the mean.

**Table 2:** Primer sequences for quantitative real-time polymerase chain reaction.

miRNA/Gene	Forward Sequences (5′-3′)	Reverse Sequences (5′-3′)
miR-145	GTCCAGTTTTCCCAGGAATC	AGGTCCAGTTTTTTTTTTTTTTTAGG
miR-16	CGCAGTAGCAGCACGTA	CAGTTTTTTTTTTTTTTTCGCCAA
*GOLM1*	TGGCCTGCATCATCGTCTTG	CCCTGGAACTCGTTCTTCTTCA
*RTKN*	ATGTTCTCCCGAAACCACCG	TTCCTCTGCAACTCCGTGTC
*HPRT1*	GGGCGGATTGTTGTTTAACTTG	GGGAACTGCTGACAAAGATTCA

## Data Availability

The authors will freely release all data underlying the published paper upon direct request to the corresponding author.
